# Viral trans-factor independent replication of human papillomavirus genomes

**DOI:** 10.1186/1743-422X-7-123

**Published:** 2010-06-10

**Authors:** Daraporn Pittayakhajonwut, Peter C Angeletti

**Affiliations:** 1 Nebraska Center for Virology, School of Biological Sciences, University of Nebraska Lincoln, Lincoln, NE, 68583-0900, USA

## Abstract

**Background:**

Papillomaviruses (PVs) establish a persistent infection in the proliferating basal cells of the epithelium. The viral genome is replicated and maintained as a low-copy nuclear plasmid in basal keratinocytes. Bovine and human papillomaviruses (BPV and HPV) are known to utilize two viral proteins; E1, a DNA helicase, and E2, a transcription factor, which have been considered essential for viral DNA replication. However, growing evidence suggests that E1 and E2 are not entirely essential for stable replication of HPV.

**Results:**

Here we report that multiple HPV16 mutants, lacking either or both E1 and E2 open reading frame (ORFs) and the long control region (LCR), still support extrachromosomal replication. Our data clearly indicate that HPV16 has a mode of replication, independent of viral trans-factors, E1 and E2, which is achieved by origin activity located outside of the LCR.

## Background

Papillomaviruses (PVs) infect the basal layers of epithelial cells and maintain their genomes at constant, but relatively low-copy number in basal epithelial cells. These viruses replicate their genomes as nuclear plasmids in their natural mammalian host cells. Understanding of Human papillomavirus (HPV) replication has lagged behind that of other DNA viruses due to the need for development of efficient cell culture systems [1-3]. Most of our knowledge of HPV replication is derived from extensive studies of BPV1 in established rodent cell lines (C127), since BPV1 was found to transform and replicate episomally in these cells. Short-term replication assays were performed in transformed cells, in order to identify the *cis*- and *trans*-elements that were required for replication of PVs [4]. Using this approach, with BPV1, the early proteins E1 and E2 were found to be required for viral DNA replication [5-7]. These viral proteins interact with their cognate binding sites, located within the LCR, referred to as the origin of replication (ori). More detailed analyses have shown that the minimal BPV origin includes multiple E2BSs, an E1BS, and also an AT-rich region [8].

Genetic analysis of HPV11 and 18 transient replication also suggested that both viral proteins E1 and E2, as well as the origin of replication containing one or more E2BS and putative E1BS and AT-rich region, were essential for HPV DNA replication [7,9-12]. Several experiments, including those performed by cell-free DNA replication, revealed that E1 had ATP-dependent helicase activity and recruits DNA polymerase α to the viral ori to initiate replication. Efficient replication also depends on all other DNA replication proteins; DNA polymerase δ and ⟨, proliferating cell nuclear antigen (PCNA), replication protein A (RPA), and topoisomerases I and II, that are provided by the host cell [13,14].

Although it is apparent that the minimal requirement for HPV replication is analogous to that for BPV1 in transiently transfected cells, HPV16 genomic DNA has been shown to replicate at lower efficiency than other HPVs [6]. Furthermore, results from cell free replication assays with various combinations of ori and viral proteins showed certain differences in replication between BPV and HPV. Variations in the viral replication efficiency observed with cell extracts from different sources might reflect dissimilar function of HPV with different host cellular replication machinery [14].

Published data that indicated a requirement for E1 and E2 proteins among PVs was mostly from transient transfection assays that were performed in the context of exogenously expressed viral proteins. The concentrations used in those experiments would not reflect physiological levels of the viral proteins in the basal layer of epithelial cells in natural host tissue. Since the viral life cycle is tightly linked to the differentiation status cell in the epithelial compartment, it is conceivable that HPVs may have more than one mode of DNA replication, so as to allow fine-tuning of the viral copy number. In fact, recent data has shown that an E1-independent mode of replication exists in the viral replication at an early stage of the viral life cycle [15,16]. This result is in agreement with previous experiments in *Saccharomyces cerevisiae *showing that various HPVs can support viral replication in the absence of both E1 or E2 proteins [17]. Moreover, the *cis*-acting elements required for E1 and E2 -independent replication and maintenance were also mapped outside the LCR region [18]. These findings lead us to hypothesize that at certain point of the viral life cycle, the HPV replication may not require both of the viral trans-factors, E1 and E2, but may rely solely on cellular replication machinery.

In this study, we analyzed the requirement for E1 and E2 in HPV16 DNA replication at the early stage of the viral life cycle. Using a short-term replication assay, as previously used for BPV and HPVs [5,6], we have found that HPV16 is able to replicate independently of viral trans-factors, E1 and E2. We present evidence that HPV16 possesses a distinct origin of replication-activity, located in a region outside of the viral LCR that is not recognized by the viral proteins, E1 and E2. However, replication of HPV16 DNA in the absence of heterologously expressed E1 and E2 proteins is relatively low compared to the E1 and E2-mediated replication. This is consistent with a low, maintenance level of replication. In contrast to previous results from transient transfection and cell free system [5,7], we observed species and cell-type specificity to HPV16 DNA replication under conditions in which E1 and E2 were omitted.

## Results

### Transient replication of HPV16 genomic DNA in different cell lines

**Figure 1:**
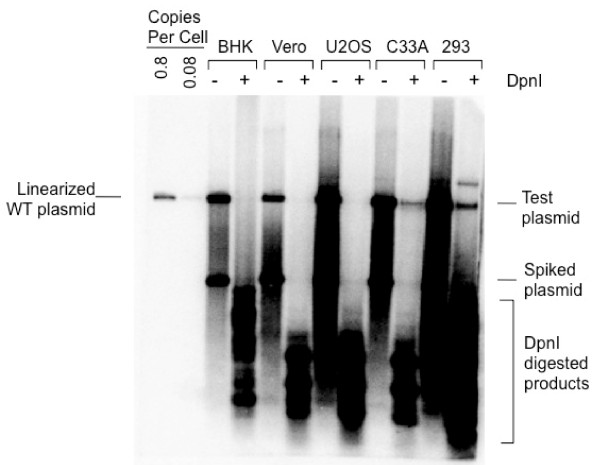
**Transient replication of HPV16 DNA in different cell types**. Five μg of plasmid containing HPV16 genomic DNA were transfected into various cell lines; baby hamster kidney cell (BHK), American green monkey kidney cell (Vero), human osteoblastoma cell (U2OS), human cervical carcinoma cell (C-33A) and human embryonic kidney cell (293). After 4 days of transfection, low molecular weight DNA was extracted from 1 × 10^7 ^cells by Hirt method. During extraction, 10 ng of pUC19 DNA was added as a "spiked plasmid" to monitor the recovery of the plasmid DNA from transfected cells and to test the completeness of DpnI digestion. For the Southern analysis, 10% of Hirt-DNA was digested with HindIII to linearize the plasmid and 90% was digested with both HindIII and DpnI. The blot was probed with random-primed^32^-P labeled pUC19 DNA. As a control, linearized plasmid containing HPV16 at concentrations of 100 and 10 pg was loaded on the left-most lanes of the blot.

In order to determine the requirement for viral trans-factors in HPV16 DNA replication, different mammalian cell lines of epithelial or fibroblast lineage were examined for the ability to support transient viral DNA replication. Baby Hamster Kidney cells (BHK), monkey kidney cells (Vero), human osteoblasts (U2OS), human cervical cells (C33A), and adenovirus 5-transformed human kidney epithelial cells (293) were transfected with 5 μg o f a plasmid containing an entire HPV16 genome. At 4 days after transfection, low-molecular-weight DNA was harvested and subjected to DpnI digestion. Input DNA propagated by DAM^+ ^bacteria is methylated at adenine bases within the GATC sequence, and is thus sensitive to DpnI digestion, whereas DNA replicated in mammalian cells loses the adenine methylation and thus, becomes DpnI-resistant. The replicated DNAs were detected as full-length plasmids by Southern analysis using pUC19 as a probe. Human 293 and C33A demonstrated the ability to support viral DNA replication at a low level, whereas BHK, Vero, and U2OS failed to replicate under the same conditions (Figure 1). Variation in viral DNA replication in different cell lines clearly indicates a degree of species and/or cell-type specificity in HPV16 replication. This also reflected the greater reliance of HPV16 on the host replication machinery when E1 and E2 were not exogenously supplemented.

### Analysis of HPV16 E1- and E2-independent mode of replication

**Figure 2:**
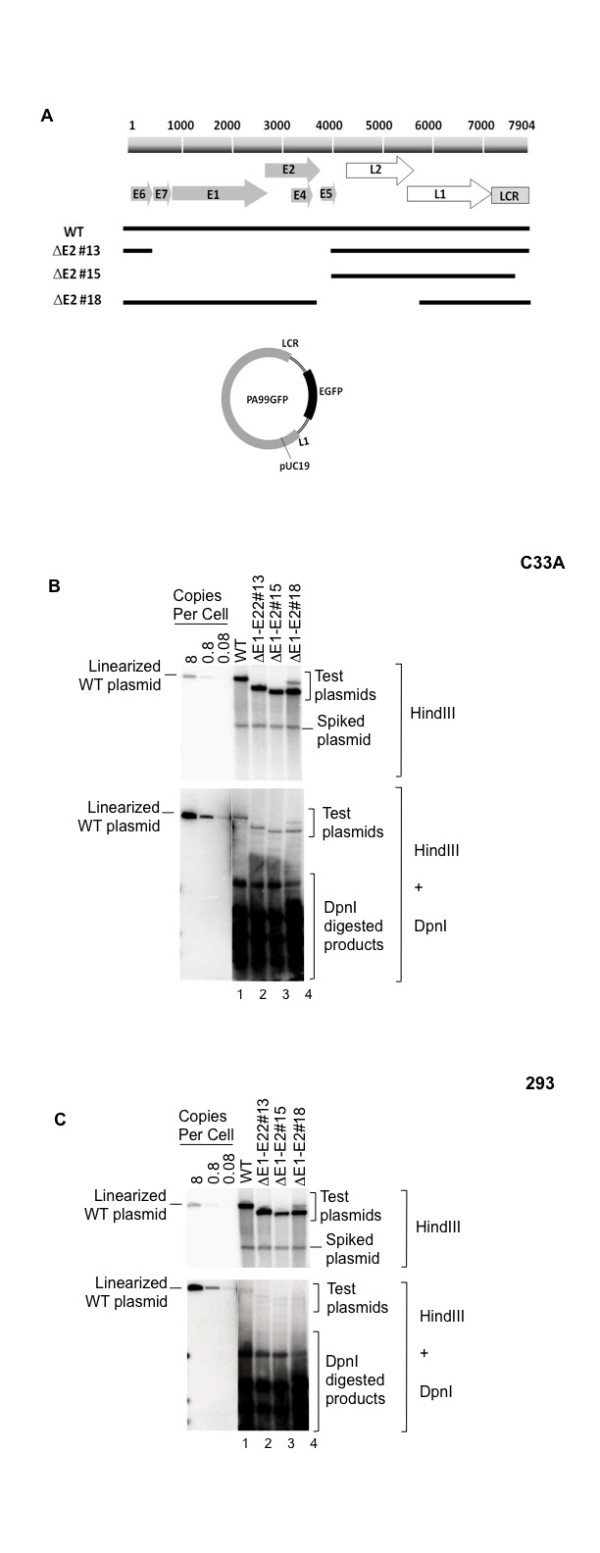
**Replication of HPV16 DNA independently of E1 and E2 proteins**. (A) Maps of PA99GFP and its mutant derivatives. The top bar represents a linear map of the HPV16 genome with responsive eight ORFs shown in arrows and the LCR indicated by a gray bar. The lines below show HPV16 segments, designated wild type (WT) and respective deletion mutants. PA99GFP contains an entire HPV16 DNA, an enhanced green fluorescent protein (EGFP) under a CMV promoter in a pUC19 backbone. The mutants ΔE1-E2-#13, ΔE1-E2#15, and ΔE1-E2#18 harboring HPV16 DNA sequenced denoted by solid bars were created from pPA99GFP as described in Materials and Methods. Autoradiograms of representative Southern blots show extrachromosomal DNA (HindIII-digested products) and replicated DNA (DpnI-resistant) from transient replication assays with WT HPV16 and mutants in C33A (B) or 293 (C). Cells were transfected with WT or mutant HPV16 DNA containing plasmids as indicated. At 4 days post-transfection, extrachromosamal DNA were isolated from 1 × 10^7 ^cells. For Southern analysis, 10% of each sample was linearized with single cutting enzyme, HindIII and 90% was digested with HindIII and DpnI. Blots were hybridized with random-primed radiolabeled pUC19 DNA. The blots with HindIII digested DNA were exposed for 2 h whereas the ones with double digestion (HindIII + DpnI) were exposed for 4 days. The pUC19 backbone plasmid was used as the "spiked plasmid" to monitor the efficiency of plasmid recovery and completeness of DpnI digestion. The control DNA shown on the left, contains 1 ng,

Based on the observation of E1-independent HPV replication in previous studies in yeast and mammalian cells, we speculated that HPV16 may not entirely require either E1 or E2 proteins for at certain early points in the viral life cycle. To investigate the necessity for E1 and E2 in HPV16 DNA replication, HPV16 mutants with deletions that included the E1 and E2 ORFs were generated in the background of the entire viral genome in a pUC19 backbone. The mutants, designated as ΔE1-E2#13 and ΔE1-E2#15 lack E1 and E2 sequences to differing extents, as shown in Figure 2A. These deletion mutants were examined for short-term replication using human epithelial cells, 293 and HPV-negative human cervical cells, C33A that were previously shown to support the replication of wild type (WT) HPV16. The transient replication assay most closely mimics the early phase of the HPV life cycle, thus, representing the initial viral DNA replication upon HPV infection. Analysis of short-term replication of HPV16 mutants defines the *cis*- elements required for HPV replication at the establishment phase of the HPV life cycle. Equal amounts of HPV16 mutants (0.6 pmole equivalent) were transfected into 1.5 × 10^6 ^cells by the liposome method. To compare replication efficiency, a plasmid containing an entire HPV16 genome was transfected in parallel under the same conditions. Low-molecular-weight DNA was extracted at 4 days post-transfection and analyzed for DpnI-resistant replicated DNA as described above.

As shown in Figure 2B and 2C, a mutant with a deletion that included the E1 and E2 ORFs, designated as ΔE1-E2#13 (lanes 2) showed DNA replication at a level comparable to that of the WT HPV16 in either 293 or C33A cells. A similar level of replication was also observed in a mutant, ΔE1-E2#15 (lane 3) that lacks both of E1-E2 coding regions and part of the LCR, which contains the E1 and E2 binding sites at the origin of replication. These results indicate that the viral proteins, E1 and E2, are not absolutely necessary for the viral DNA replication. Thus, it is likely that HPV16 has sequences outside of the LCR, which provide a mode of replication that is not dependent on viral *trans*-factors, but potentially analogous to an autonomously replicating sequence (ARS).

To determine if HPV16 carries a distinct replication activity that functions independently of E1 and E2 proteins, a series of mutants with progressive deletions spanning the LCR to the E1 and E2 ORFs were constructed and tested for ability to replicate in a transient transfection assay. The deletion mutants were created in a context of a whole HPV16 genomic DNA in a pUC19 backbone. The mutant, ΔLCR, contains a deletion (nt 7266 to 7904/1 to 269) in the LCR sequence where E1 and E2 bindings sites are located. In the ΔE1-E2 mutant, the whole E1 and E2 ORFs (nt 269 to 4466) were deleted. The mutant, ΔLCR-E2, contains a combination of deletions in the ΔLCR and ΔLCR-E2 mutants, whereas the L1 mutant contains only a 3'-segment of the L1 ORF (nt 6156 to 7265). The replication activities of these mutants were analyzed in both C33A and 293 cells.

**Figure 3:**
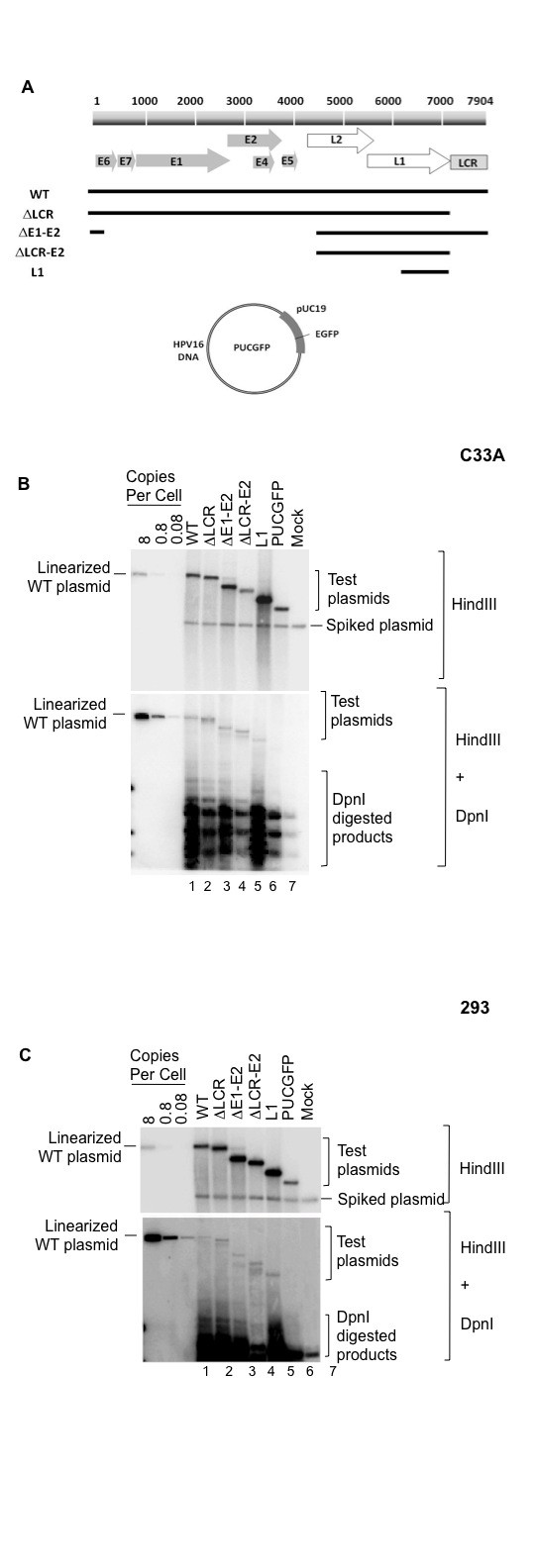
**Transient replication of HPV16 mutants with LCR and E1-E2 deletions**. (A) Schematic representation of PUCGFP comprising an EGFP in a pUC19 backbone. An entire sequence and partial fragments of HPV16 DNA shown in solid lines were inserted into PUCGFP at BamHI to generate WT and mutants designated ΔLCR, ΔE1-E2, ΔLCR-E2, and L1 as described in Materials and Methods. Replication efficiencies of the HPV16 WT and mutants are evaluated by a transient transfection assay. Autoradiograms of representative Southern blots show replicated DNAs from transient transfection assays in C33A (B) and 293 (B). The assay was performed as described in Figure 2 legend. Ten pg to 1 ng of linear plasmid containing the entire HPV16 DNA, was loaded on the left-most lanes, as copy number controls.

When we performed a transient transfection assay, each mutant was found to replicate at a similar level to that of WT HPV16 (Figure 3B and 3C, lanes 2 to 5), whereas, the plasmid backbone without HPV16 DNA sequences, PUCGFP, did not show any detectable DNA replication in either 293 and C33A cells (Figure 3B and 3C, lane 6). Interestingly, the mutant containing only a partial segment of L1 coding region was able to replicate in both cell lines with efficiency comparable to those of WT and other mutants. These observations not only confirm the existence of E1 and E2 independent mode of replication in HPV16 but also indicate that the virus has a distinct replication function, outside of the LCR, the activity of which is likely to be mediated only by cellular replication machinery. Our data indicate that a viral-trans-factor independent *cis*-replicating element may reside in the late region of HPV16 genome (L2-L1 ORFs).

## Discussion

HPVs establish their life cycle in the stratified epithelium of skin or mucosa. The virus replicates and amplifies its genome in two different stages, non-productive and productive cycles. In the non-productive stage, the first amplification replication occurs immediately after infection and the viral DNA is stably maintained in the undifferentiated basal cells. Whereas the second amplification replication, which increases the HPV DNA copy number to several hundreds or thousands per cells, takes place after differentiation of the host cell. Based on the knowledge acquired by studies with BPV1, the early step in the PV replication may occur at relatively low levels of E1 and E2 proteins. This amplification is thought to be rapid and transient, after which, the viral DNA is maintained at low copy number during the maintenance phase and persists in a latent stage.

It would be reasonable to suggest that during maintenance phase, HPV keeps the levels of E1 and E2 and the viral copy number low in the infected basal cells, so as to evade cellular immune surveillance. It is also logical that the virus may employ a different mode of replication, which relies more on cellular replication machinery to stably retain the genome as a nuclear plasmid in the proliferating basal epithelial compartment. The virus replicates once per cell generation in the presence of low-levels of E1, and later converts to random-choice replication, with multiple rounds of viral DNA replication per cell generation, when E1 is highly expressed [19]. This concept is also consistent with results of previous studies which found that E1 was dispensable for PV replication, under some conditions [15].

There are conflicting results concerning the requirement of E2 in replication of PVs. In some studies, E2 ORF has been shown not to be necessary for replication while others have found an absolute requirement of E2 [6,20,21]. The role of E2 in replication is not entirely clear. E2 has been found to alleviate nucleosomal-mediated repression of replication by altering the chromatin structure around the ori, thus allowing accessibility of cellular replication factors [22]. It also directs E1 to bind to the ori DNA (which it normally has only low specificity for), this cooperation leads to activation of DNA replication [23]. However, this function can be bypassed if E1 is expressed at high levels [24]. Therefore, it appears that E2 plays more of a facilitory role in E1-dependent DNA replication.

In our previous study, we mapped the cis-acting replication and maintenance signals in the HPV16 genome that are required for long-term stability [25]. Those studies revealed that cis-elements in the late region (L2-L1 ORFs) function in E2-independent genome maintenance. The same studies led us to hypothesize that E1-E2-independent replication function also resides within the same region of the HPV genome. In order to assess viral trans-factor independent replication apart from other influences, we chose to do short-term replication assays described in the present study.

In this study, we report the autonomous replication of HPV16 containing plasmids in the absence of E1 and E2 ORFs or their protein products (Figures 1, 2 and 3). The E1 and E2 ORF mutants were able to replicate at a level comparable to WT. Similar results were obtained from transient replication assays using HPV16 genome that contains a deletion of the LCR and progressive deletions extending from the LCR to E2 coding region. The level of viral trans-factor-independent replication, is clearly above background, thus, we conclude that it represents an intrinsic activity of the HPV genome, separate from the E1-dependent ori. Since E1 is a helicase that plays a role in replication by recruiting DNA polymerase α and unwinding DNA, we infer that such functions must be substituted by cellular helicases, such as mini-chromosome-maintenance proteins (MCMs), and Werner and Bloom helicases.

**Figure 4:**
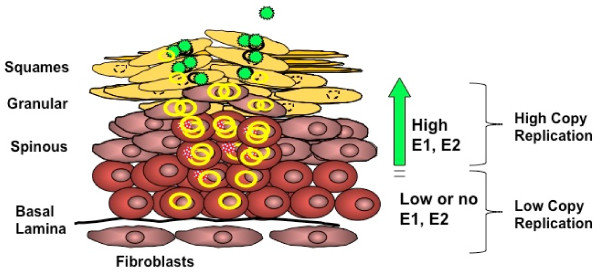
**A Model regulation of HPV copy number during the viral lifecycle**. The yellow circle represents the viral episomes. During the maintenance phase, expression of E6 and E7 induces proliferation of basal keratinocytes and the viral genome is replicated extrachromosomally at low-copy number (1-10 copies per cell). Little if any E1 and E2 protein is expressed in the basal layer cells, which leads to theta-form replication of HPV genomes. As the cells differentiate, expression of E1 and E2 trans-factors increases in the spinous layer. A transition from theta to rolling circle replication results in an increase in copy number up to 100-1000 copies per cell. Post-amplification, high levels of L1 and L2 capsid genes are expressed and capsid assembly occurs in the granular and squamous layers of the stratified epithelium. Progeny virus is released by desquamation.

E1-E2 independent replication may contribute to a true form of latency, in which the virus minimizes its expression of viral proteins in host cells and keeps the viral copy number low by utilizing cellular replication proteins under S phase control (Figure 4). A mechanism of DNA replication that is controlled by the cell has been well studied in EBV, whose DNA is replicated one time per S phase in latently infected cells [26,27]. This mode of replication is achieved through replication licensing proteins, MCMs an ORC, which are restricted to late mitosis and G1 phase of cell cycle [28]. These proteins assemble on the latent origin of replication, OriP, of EBV and are also known to complex with the latent origin of replication of KSHV DNA [29]. The low level of viral genome replication, as regulated by cellular factors, is a beneficial and common strategy for episomal viruses to establish latency. We suggest that HPV may adopt this mechanism to control its genome replication in favor of DNA maintenance during its persistence phase in the host. Based on the similar magnitude of WT versus E1/E2 ORF mutants, our results suggest that E1-E2-independent replication may be the preferred mode of replication upon the initial infection and that higher levels of E1 alter the mode of replication later in the infectious process. However, we cannot presently exclude the possibility that viral-trans-factor independent replication occurs throughout the viral lifecycle, even at the same time as E1-dependent replication is ongoing.

To date, it is not clear whether the cellular DNA replication licensing proteins are associated or necessary for replication in the absence of E1 and E2 with HPV16 DNA. It is interesting that E1, which is an ATP-dependent helicase and origin-binding of HPV, has many similarities to cellular MCM proteins. In fact, recent preliminary work has shown that E1 directly interacts with MCM2 and MCM6 of the MCM hexamer (Kenneth Alexander, personal communication). Since this binding is specific, it is likely that E1 and MCMs share conserved structures that are interchangeable to form a hybrid-helicase hexamer at the origin of replication. This possibility is currently under investigation. Our studies suggest that E1-E2 independent replication may act to preserve a latent-like state where MCMs are substituted for E1 and replication is carried out by cellular proteins that control the replication in once-per-cell cycle manner [19]. This strict replication is overcome by expression of E1 that switches replication to a multiple DNA copy per-cell-cycle mode, perhaps by directly modifying MCM complexes.

Under conditions in which E1 and E2 were not expressed exogenously, WT HPV16 and all mutants, exhibited low-copy replication in human cervical cells (C33A) and human embryonic kidney cells (293). This result is in contrast to experiments in which ectopic expression of E1 and E2 was provided, in which a wide range of mammalian cells were reported to support LCR-dependent replication [6,7,14]. Cell and species specificity in viral replication have been reported in the other members of the papovavirus family; SV40 and polyomavirus replication specificities rely on species-specific interactions between large tumor antigen and the cellular DNA polymerase α/primase complex and the single-stranded DNA binding protein [30-32]. It is likely that the replication restriction also exists for papillomaviruses. As results demonstrated in this and previous studies [33,34], variation of cell and tissue restriction for HPV replication suggests that HPVs may rely to a greater degree on specific cellular factors than previously thought. This may explain the strict host range of these viruses, which is, in part, implicated by the presence of cellular factors necessary for the initiation of DNA replication.

Our study demonstrates that E1-E2-independent HPV DNA replication occurs by an alternative mechanism directed to sites outside of the LCR. The replication efficiency is relatively low and cell-type restricted, which makes sense for a virus which spends much of its effort to avoid immune surveillance. It is speculated that cellular factors may play a dominant role in this mode of replication. Potential mechanisms that may account for E1-E2-independent replication and maintenance may include recruitment of transcription factors and/or recruiting of cellular replication machinery by cellular proteins. Our recent results suggest that cellular chromosomal segregation (centromeric, metaphase chromatin, and telomeric) regulation proteins are reasonable candidates for this role. Further dissection of the cis-and trans-acting components that regulate E1-E2-independent HPV replication is required to understand its role in the context of the viral life cycle.

## Methods

### Mammalian cells and transfection methods

American green monkey kidney; Vero, osteoblastoma; U2OS, human embryonic kidney; 293, and human cervical carcinoma cells; C33A were grown in Dulbecco's modified Eagle's medium (DMEM) supplemented with 10% fetal bovine serum and 1 mM sodium pyruvate in a 5% CO_2 _incubator at 37°C. Baby hamster kidney; BHK cells, were cultured in DMEM supplemented with 5% fetal bovine serum. All transfections were performed with eqimolar amounts of plasmid DNA by the use of Dreamfect reagent (Ozbiosciences). Cells were seeded at the density of 1.5-2 × 10^6 ^cells in a 100 mm plate for 16 to 24 h prior to transfection.

### Plasmids and library constructions

The PA99GFP plasmid contains a full-length HPV16 genome cloned into pUC19 with enhanced green fluorescent protein (EGFP) under the regulation of the cytomegalovirus promoter. This plasmid was modified from pPA99 [17] by insertion of EGFP gene which was PCR-amplified from pGFP C1 using primers (5' primer: 5'-GCATC**CTCGAG**GTAATCAATTACGGG-3' and 3' primer: 5'-CGAAGCTTGAG**CTCGAG**ATCTGAG-3') containing XhoI site (bolded) into the XhoI site of pPA99. Deletion mutants designated ΔE1-E2#13, ΔE1-E2#15 and ΔE1-E2#18 which are indicated in Figure 2A, were obtained from modifications of PA99GFP. The HPV16 E2 deletion mutants were made by cleavage of PA99GFP with SexAI and XcmI. After the removal of SexAI-XcmI fragment, the protruding ends were subsequently repaired by DNA polymerase I (Klenow) and re-ligated. The resultant plasmids were screened for ones that lack the SexAI-XcmI fragment but retain the selectable marker. Three plasmids were selected and subjected to DNA sequencing analysis. The first one had a 3585 bp deletion (nucleotides 339-3924) resulting in removal of the E1 and E2 ORFs referred to as ΔE1-E2#13. The second one, named ΔE1-E2#15, lacked HPV16 sequences between nucleotides 7394-7904 and 1-3924 within the LCR, E6, E7, E1 and E2 ORFs. For the last HPV16 E2 mutant, designated as ΔE1-E2#18, a deletion was created between nucleotides 3661-5240, thus leaving E1 ORF intact but truncating the E2 and L2 ORFs.

The HPV16GFP plasmid contains the entire HPV16 genome in pUCGFP that carries an expression cassette of EGFP gene in a pUC19 backbone. HPV16 mutants designated ΔLCR-E2, ΔE1-E2, and L1 were derived from HPV16GFP as previously described [25]. The mutant ΔLCR was made by deleting a 909-bp fragment between PmlI and BsaBI sites and then re-ligated by T4 DNA ligase. To confirm the deletion, DNA sequencing analysis was performed afterward by standard procedures. A map of HPV16GFP and its derivative constructs is shown in Figure 3A.

### Short-term DNA replication assays

Cells were seeded into a 100 mm dish at the density indicated above and transfected with 0.6 pmoles of plasmid DNA using the manufacturer's instructions. The plasmid-containing cells were grown under non-selective conditions for 4 days prior to analysis. Plasmid DNA was isolated using the Hirt method [35] from equal numbers of cells (6 × 10^6 ^- 1 × 10^7 ^cells). Ten ng of pUC19, referred to as "spiked DNA", was added to each sample prior to cell lysis in order to monitor the efficiency of plasmid isolation from cells and the completeness of subsequent DpnI digestion. Approximately 10% of each sample was digested with 10 U of a single-cutting enzyme, HindIII, at 37°C for 4 h, (resulting in over 90% completion of HindIII digestion), followed by overnight digestion with 20 U DpnI. Upon digestion, DNA was separated on a 0.8% agarose gel and then transferred to a nitrocellulose membrane. Blots were hybridized at 42°C overnight with a pUC19 DNA probe radiolabeled by random priming kit (GE Healthcare). Autoradiography was carried out by use of a PhosphoImager (Bio-Rad) and quantified by the Quantity One program (Bio-Rad). Relative replication activities were quantified from a phosphorimage of the Southern blots. The percentages of replicated DNA refer to the ratio of the level of replicated DNA (DpnI-resistant plasmid) over the level of HindIII-linearized plasmid. The numbers were normalized against the level of the spiked DNA in each sample.

## List of Abbreviations

HPV: Human Papillomavirus; LCR: Long Control Region; Ori: Origin of Replication; MCM: Minichromosome Maintenance Proteins; DNA: Deoxyribonucleic Acids; EGFP: Enhanced-Green-Fluorescent Protein.

## Competing interests

The authors declare that they have no competing interests.

## Authors' contributions

The authors contributed equally to this manuscript. All authors read and approved the final manuscript.
